# Differential expression of cell cycle and WNT pathway-related genes accounts for differences in the growth and differentiation potential of Wharton’s jelly and bone marrow-derived mesenchymal stem cells

**DOI:** 10.1186/s13287-017-0555-9

**Published:** 2017-04-26

**Authors:** Aristea K. Batsali, Charalampos Pontikoglou, Dimitrios Koutroulakis, Konstantia I. Pavlaki, Athina Damianaki, Irene Mavroudi, Kalliopi Alpantaki, Elisavet Kouvidi, George Kontakis, Helen A. Papadaki

**Affiliations:** 1 University of Crete School of Medicine, Heraklion, Greece; 2 Graduate Program “Molecular Basis of Human Disease”, University of Crete School of Medicine, Heraklion, Greece; 3 Department of Obstetrics and Gynecology, University of Crete School of Medicine, Heraklion, Greece; 4 Department of Orthopedics and Traumatology, University of Crete School of Medicine, Heraklion, Greece

**Keywords:** Bone marrow, Wharton’s jelly, Mesenchymal stem cells, Cell cycle, WNT pathway

## Abstract

**Background:**

In view of the current interest in exploring the clinical use of mesenchymal stem cells (MSCs) from different sources, we performed a side-by-side comparison of the biological properties of MSCs isolated from the Wharton’s jelly (WJ), the most abundant MSC source in umbilical cord, with bone marrow (BM)-MSCs, the most extensively studied MSC population.

**Methods:**

MSCs were isolated and expanded from BM aspirates of hematologically healthy donors (n = 18) and from the WJ of full-term neonates (n = 18). We evaluated, in parallel experiments, the MSC immunophenotypic, survival and senescence characteristics as well as their proliferative potential and cell cycle distribution. We also assessed the expression of genes associated with the WNT- and cell cycle-signaling pathway and we performed karyotypic analysis through passages to evaluate the MSC genomic stability. The hematopoiesis-supporting capacity of MSCs from both sources was investigated by evaluating the clonogenic cells in the non-adherent fraction of MSC co-cultures with BM or umbilical cord blood-derived CD34^+^ cells and by measuring the hematopoietic cytokines levels in MSC culture supernatants. Finally, we evaluated the ability of MSCs to differentiate into adipocytes and osteocytes and the effect of the WNT-associated molecules WISP-1 and sFRP4 on the differentiation potential of WJ-MSCs.

**Results:**

Both ex vivo-expanded MSC populations showed similar morphologic, immunophenotypic, survival and senescence characteristics and acquired genomic alterations at low frequency during passages. WJ-MSCs exhibited higher proliferative potential, possibly due to upregulation of genes that stimulate cell proliferation along with downregulation of genes related to cell cycle inhibition. WJ-MSCs displayed inferior lineage priming and differentiation capacity toward osteocytes and adipocytes, compared to BM-MSCs. This finding was associated with differential expression of molecules related to WNT signaling, including *WISP1* and *sFRP4*, the respective role of which in the differentiation potential of WJ-MSCs was specifically investigated. Interestingly, treatment of WJ-MSCs with recombinant human WISP1 or sFRP4 resulted in induction of osteogenesis and adipogenesis, respectively. WJ-MSCs exhibited inferior hematopoiesis-supporting potential probably due to reduced production of stromal cell-Derived Factor-1α, compared to BM-MSCs.

**Conclusions:**

Overall, these data are anticipated to contribute to the better characterization of WJ-MSCs and BM-MSCs for potential clinical applications.

## Background

In the past 16 years, the field of mesenchymal stem (stromal) cells (MSCs) has progressed at a great pace, as their exceptional characteristics are being unraveled and encouraging data from preclinical and clinical studies accumulate. In this regard, MSC-based treatment is now considered as a promising modality in the therapeutic intervention of various diseases or tissue damage [1].

Bone marrow (BM)-derived MSCs have been considered as the gold standard for clinical applications. However, there is currently great interest in identifying alternative, more easily accessible sources of MSCs. In this context, umbilical cord (UC), a medical waste obtained with a painless, simple and safe procedure during delivery, has drawn much attention as an attractive substitute of the BM for MSC isolation [2].

MSC origin may have an important impact in the biological properties of the cells and may even affect their effectiveness in cell-based therapies [3]. As regards to differences between BM- and UC-derived MSCs, contradictory results have been published so far, probably due to discrepancies in the isolation protocols of UC-MSCs in different studies [4]. Interestingly, MSCs from different UC compartments have been reported to possess different biological properties [5].

In view of the current interest in exploring the use of UC-MSCs from different sources in the clinic, the proper characterization of the cells is of particular importance. In the present study, we have performed a side-by-side comparison of MSCs isolated from the Wharton’s jelly (WJ), the most abundant source of MSCs in UC [4], with BM-MSCs, the most extensively studied adult MSC population [3]. Specifically, we have comparatively studied the biological properties of the WJ-MSCs and BM-MSCs, namely their proliferative potential, the survival and senescence characteristics, the differentiation potential toward osteocytes and adipocytes and their capacity to support hematopoiesis. We have also explored the cell cycle distribution of the two MSC populations, in association with the expression of cell cycle-related genes. Because the WNT pathway has been involved in the proliferation and differentiation process of MSCs from different sources, we have probed the expression of genes related to the WNT signaling and the effect of WNT-related molecules in the differentiation capacity of WJ-MSCs. The results of the study have pointed out significant differences in the biological properties of BM- and WJ-derived MSCs that should be taken into consideration when choosing the best MSC source for a given application.

## Methods

### Samples

UC samples were obtained from 18 full-term neonates (38 to 39 weeks) following normal labor. BM samples were obtained from 18 hematologically healthy individuals (nine males and nine females, median age 50 years) undergoing orthopedic surgery for hip replacement. The study has been approved by the Ethics Committee of the University Hospital of Heraklion and informed consent for the UC and the BM donation was obtained from the neonate mothers and the individuals undergoing surgery, respectively, according to the Helsinki Protocol.

### MSC cultures

MSCs were isolated from BM and WJ as previously described and expanded in appropriately supplemented alpha-Minimum Essential Medium (α-MEM; Gibco Invitrogen, Paisley, Scotland), at 37 °C/5% CO_2_ fully humidified atmosphere [6–8]. The culture medium was replaced twice per week and on 80–90% confluency, MSCs were detached using 0.25% Trypsin–1mM EDTA (Gibco, Invitrogen), reseeded at a concentration of 2000 cells/cm^2^ and further expanded for a total of ten passages (P) [6, 7].

Cell-free supernatants from WJ- and BM-MSC cultures at P2, P6 and P10 were stored at -80 °C for quantification of FMS-like tyrosine kinase 3 ligand (FLT3L; Quantikine, R&D Systems, Minneapolis, MN, USA), Granulocyte- Colony Stimulating Factor (high sensitivity human G-CSF; Quantikine, R&D Systems), Stromal Cell-derived Factor-1α (human CXCL12/SDF-1α; Quantikine, R&D Systems) and Granulocyte Macrophage-Colony Stimulating Factor (human GM-CSF; Affymetrix, eBioscience, San Diego, CA, USA), by means of enzyme-linked immunosorbent assay.

### Immunophenotype and survival characteristics of MSCs

Trypsinized MSCs from P2 to P10 were immunophenotypically characterized by flow cytometry using anti-human monoclonal antibodies (mAb) against CD29 (4B4; Cyto-Stat/Beckman Coulter, Fullerton, CA, USA), CD44 (J173; Immunotech/Beckman Coulter, Marseille, France), CD73 (AD2; BD Biosciences Pharmingen, San Diego, CA, USA), CD90 (F15.42; Immunotech/ Beckman Coulter), CD105 (SN6; Caltag, Burlingame, CA, USA), anti-CD146 (P1H12; BD Biosciences Pharmingen), CD45 (IMMU19.2; Immunotech/ Beckman Coulter), CD14 (RMO52; Immunotech/Beckman Coulter), CD34 (QBend10; Beckman Coulter), CD31 (5.6E; Immunotech/Beckman Coulter), CD19 (J3-119; Immunotech/Beckman Coulter) and HLA-DR (Immu-357, Immunotech/Beckman Coulter), as previously described [6].

Flow cytometry was also used to study the apoptotic characteristics of MSCs at two representative passages (P2 and P6) by means of 7-aminoactinomycin D staining (7-AAD; Calbiochem-Novabiochem, Nottingham, UK) as previously described [9]. Results were expressed as proportions of 7-AAD^neg^ (live), 7-AAD^dim^ (early apoptotic) and 7-AAD^bright^ (late apoptotic) cells. Acquisition and analysis were performed in a Cytomics FC 500 flow cytometer (Beckman Coulter, Brea, CA, USA) on a minimum of 10,000 events.

### Cell cycle analysis of MSCs

For cell cycle analysis, BM- and WJ-MSCs from P2, at a confluency of 80–90% were trypsinized, washed with PBS and fixed with 70% cold ethanol. Cells were treated with 0.5 mg/mL RNase A (PureLink RNase A; Invitrogen) and stained with 50 μg/mL propidium iodide (PI, Invitrogen) in the dark for 30 min at 37 °C. DNA content was determined by flow cytometry and the percentage of cells in different phases of cell cycle was assessed. A minimum of 100,000 events were acquired per sample.

### Proliferative potential of MSCs

The proliferative potential of MSCs was evaluated by a 3-[4,5-dimethylthiazol-2-yl]-2,5 diphenyl tetrazolium bromide (MTT)-based assay at P2 and by estimating the population doubling (PD) time through P2-P10 [6, 7, 10]. The formula 2^n^ = Nx/No was used to calculate the PDs of cells at each passage based on the number of cells counted in the flask after trypsinization (Nx) and the number of cells initially plated (No) [6, 7, 10]. Cumulative PDs were also calculated as previously described [11].

### Assessment of MSC senescence

#### Evaluation of senescence-associated gene expression

A real-time reverse transcription polymerase chain reaction (RT-PCR) was used for the evaluation of the expression of genes associated with cell senescence, namely the senescence-associated cyclin-dependent kinase (CDKN) inhibitors *CDKN1A* (*p21*), *CDKN2A* (*p16*) and *CDKN2B* (*p15*), the tumor suppressors *TP53* and retinoblastoma (*RB1*), and the phosphate-associated RhoGAP protein-tyrosine (*PARG1*) in P2, P6, and P10 MSCs, as previously described [6, 7, 10]. Glyceraldehyde-3-phosphate dehydrogenase (*GAPDH*) was used as internal control gene and gene expression values were expressed as 2^-ΔCt^, where ΔCt = Ct ^gene of interest^ – Ct ^GAPDH^.

#### MSC relative telomere length measurement

DNA was extracted from P2, P6, and P10 MSCs using the QIAamp DNA mini kit (Qiagen, Hilden, Germany) and the relative telomere length was evaluated using a previously described semi-quantitative real-time PCR method with β-globin as control single-copy gene [12]. The relative telomere length was expressed as 2^-ΔCt^ (ΔCt = Ct ^telomere^ – Ct ^β-globin^).

#### Senescence-associated β-galactosidase (SA-β-gal) expression

Expression of SA-β-gal at P2, P6 and P10 MSCs was determined by a chromogenic assay as previously described [7, 13]. The number of SA-β-gal-positive MSCs was evaluated under a phase-contrast microscope per 100 consecutively counted MSCs. All experiments were performed in triplicate.

### MSC differentiation assays

Trypsinized WJ- and BM-MSCs from P2 were induced to differentiate into adipocytes and osteocytes as previously described [6, 10, 14]. Adipogenesis was assessed by Oil Red O and osteogenesis by Alizarin Red and Von Kossa staining [6, 14]. Furthermore, total RNA from BM- and WJ-MSCs following differentiation at P2 toward adipocytes and osteocytes was isolated, reverse transcribed and subsequently amplified by real-time RT-PCR, for the quantification of genes related to adipogenesis, namely peroxisome proliferator activated receptor-γ (*PPARG*) and CCAAT/enhancer-binding protein alpha (*CEBPA*), and osteogenesis, namely alkaline phosphate (*ALP*), osteocalcin (*OSC*), distal-less homeobox protein 5 (*DLX5*) and runt-related transcription factor 2 (RUNX2). *GAPDH* was used as internal control gene. The primer sequences and real-time RT-PCR detailed conditions have been described previously [12].

In a set of experiments, adipogenesis- or osteogenesis-related gene expression in WJ-MSCs was evaluated by real-time RT-PCR following differentiation of P2 cells in the presence or absence of 20 nM recombinant human (rh)-secreted frizzled related protein 4 (rh-sFRP4, R&D Systems) or 50 ng/ml rh-WNT1-inducible-signaling pathway protein 1 (rh-WISP1, R&D Systems), respectively.

### Cytogenetic analysis of MSCs

Conventional cytogenetic analysis of BM- and WJ-MSCs was performed at P2, P6 and P8 as previously described [15–17]. MSC metaphases were identified using trypsin-Giemsa (GTG) banding and 15 to 25 metaphase cells were analyzed and classified according to the International System for Human Cytogenetic Nomenclature [16]. A chromosomal aberration was defined as clonal abnormality when at least two metaphases were demonstrating the same structural rearrangement or chromosome gain, whereas a chromosome loss had to be identified in at least three metaphases [15, 16].

### WNT signaling pathway and cell cycle PCR arrays

Total RNA was isolated from BM-MSC (*n* = 6) and WJ-MSC (*n* = 6) cultures at P2 as previously described [12]. By using the human WNT Signaling Pathway RT^2^ Profiler™ PCR Array (SABiosciences, Qiagen) we profiled the expression of 84 genes related to WNT-mediated signal transduction. Similarly, using the human Cell Cycle RT^2^ Profiler™ PCR Array (SABiosciences, Qiagen) we profiled the expression of 84 genes related to cell cycle signaling pathway. The fold change (FC) for each gene between the group of WJ-MSCs and the group of BM-MSCs was calculated with the ΔΔCt method (FC = 2^-ΔΔCt^). At least a two-fold difference in gene expression between WJ- and BM-MSCs was considered significant [12].

#### Validation of the WNT PCR array results by real-time RT-PCR

To validate the PCR - array results, significantly down- and over-expressed genes were further assessed in BM- and WJ-MSCs (P2) by real-time RT-PCR (SYBR GreenER qPCR Supermix; Invitrogen), using the specific primers of the PCR array for *WISP1*, *sFRP4* and *GAPDH* (SABiosiences, Qiagen). Reactions were performed in Rotor-Gene 6000 using a two-step cycling program consisting of 45 cycles of 95 °C for 3 seconds and 60 °C for 30 seconds. A melting curve (62–95 °C) was generated at the end of each run to verify specificity of the reactions.

### Evaluation of the hematopoiesis-supporting capacity of MSCs

A previously described two-stage culture procedure was used to test the capacity of WJ- and BM-MSCs to support normal hematopoiesis [12]. In brief, confluent MSC stromal layers from WJ and BM samples, grown in 25cm^2^ flasks, were irradiated (10 Gy), recharged with immunomagnetically sorted (Miltenyi Biotec, Bergisch Gladbach, Germany) normal allogeneic BM- or UC blood (UCB)-derived CD34^+^ cells (5 × 10^4^) and kept in 10 mL appropriately supplemented Iscove’s modified Dulbecco’s medium (Invitrogen) at 37 °C/5% CO_2_ fully humidified atmosphere. At weekly intervals for a total of 3 weeks, cultures were fed by demi-depopulation and the non-adherent cells (NACs) were counted and assayed for clonogenic progenitor cells, namely granulocyte colony-forming units (CFU-G), macrophage CFU (CFU-M), granulocyte-macrophage CFU (CFU-GM), and erythroid - CFU (CFU-E), as previously described [12, 18, 19]. The colonies were finally defined as total myeloid, that is, total CFU-GM (CFU-G plus CFU-M plus CFU-GM), CFU-E, and total CFU (total CFU-GM plus CFU-E) [12, 18, 19].

### Statistical analysis

Data were analyzed using the GraphPad Prism Statistical PC program (GraphPad Software, San Diego, CA, USA). Grouped data were expressed as mean ± 1 standard deviation and compared by means of the non-parametric Mann–Whitney *U* test. The two-way analysis of variance was used to define differences between WJ-MSCs and BM-MSCs in PD time, gene expression and cytokine levels through passages as well as in CFU numbers in culture supernatants time course and in optical density obtained by MTT at P2. The chi-square test was used for the evaluation of differences between WJ-MSCs and BM-MSCs in the frequency of cytogenetic aberrations through passages.

## Results

### BM- and WJ-MSCs exhibit similar morphologic and immunophenotypic characteristics

BM- and WJ-derived MSCs were successfully expanded and serially reseeded for ten passages. Cultured MSCs from both sources displayed the characteristic spindle-like morphology and the immunophenotypic analysis throughout P2-P10 demonstrated that cultures constituted of a homogenous cell population positive for CD73, CD90, CD146, CD105, CD29, CD44 and negative for CD31, CD19, CD45, CD14, CD34 and HLA-DR surface antigens. No difference was identified between BM- and WJ-MSCs in the expression of any of the above markers.

### BM-MSCs grow at a slower rate compared to WJ-MSCs

**Fig. 1:**
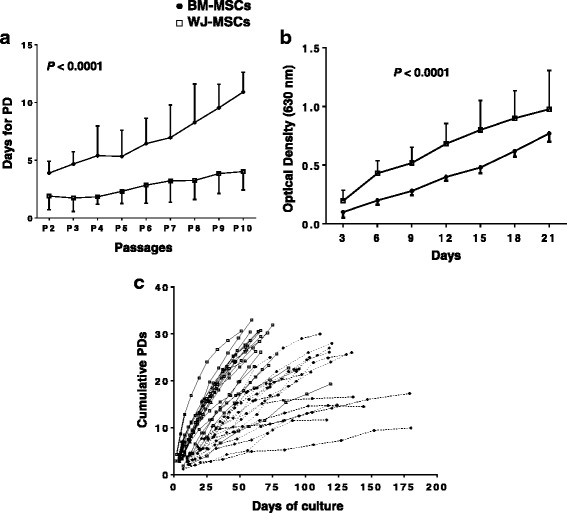
Growth characteristics of BM-MSCs and WJ-MSCs. Graph **a** depicts the days (mean ± 1standard deviation) for population doubling (PD) of BM-MSCs (n = 18) and WJ-MSCs (n = 18) through passages (*P*). Graph **b** depicts the results of the optical density (mean ± 1 standard deviation) corresponding to the number of metabolically active cells over a 21-day culture using the MTT assay in MSCs at P2. Graph **c** depicts the individual long-term growth curves of BM-MSC and WJ-MSC cultures. Every cell passage is indicated by a point and the number of cumulative PDs was calculated based on the ratio of cells seeded versus cells harvested per passage. Comparison between BM- and WJ-MSCs for PD and MTT was performed by the two-way analysis of variance. Abbreviations: *BM-MSCs* bone marrow-mesenchymal stem cells, *MTT* 3-[4,5-dimethylthiazol-2-yl]-2,5 diphenyl tetrazolium bromide, *WJ-MSCs* Wharton’s jelly-mesenchymal stem cells

The PD time in BM-MSC cultures was significantly increased in comparison to WJ-MSCs throughout P2-P10. Specifically, as shown in Fig. 1a, the PD time ranged from 3.88 ± 1 days (P2) to 10.9 ± 1.7 days (P10) in BM-MSCs versus 1.88 ± 1.18 days (P2) to 4 ± 1.6 days (P10) in WJ-MSCs (*P* < 0.0001). In accordance with these findings are the results obtained by the MTT assay at a representative passage (P2), which showed that the number of metabolically active BM-MSCs, corresponding to the obtained optical density, remained significantly lower during the 21-day culture period, compared to WJ-MSCs (*P* < 0.0001) (Fig. 1b). Finally, the individual long-term growth curve data depicted in Fig. 1c, clearly show the inferior proliferative potential of BM-MSCs compared to WJ-MSCs. Taken together, these findings suggest that BM-MSCs grow at a significant slower rate compared to WJ-MSCs.

### BM- and WJ-MSCs display similar survival characteristics through passages

To investigate whether the difference in the growth rate between BM- and WJ-MSCs is associated with altered survival characteristics, we evaluated the proportion of apoptotic cells at P2 and P6 by means of 7-AAD expression. No statistically significant differences were identified, however, in the proportion of apoptotic cells (expressed as the sum of 7-AAD^dim^ and 7-AAD^bright^ cells) between BM- and WJ-MSCs at either P2 (8.42 ± 2.6% and 7.9 ± 1.92%, respectively) or P6 (7.7 ± 4.66% and 6 ± 3.25%, respectively).

**Fig. 2:**
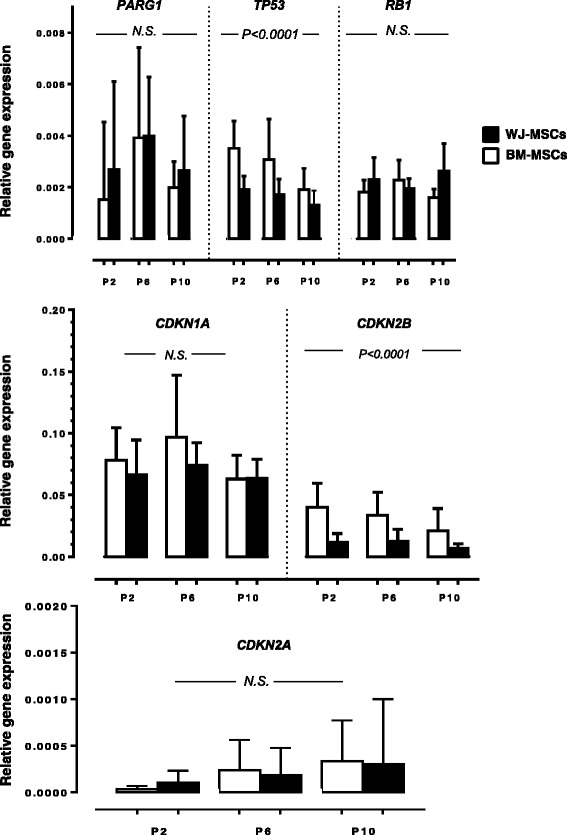
Senescence-associated gene expression in BM-MSCs and WJ-MSCs. The *bars* represent the relative expression (mean + 1 standard deviation) of the senescence-associated genes *PARG1*, *TP53*, *RB1*, *CDKN1A*, *CDKN2A*, and *CDKN2B* in BM-MSCs (n = 18) and WJ-MSCs (n = 18) through passages (*P*). Gene expression analysis was performed by means of real-time RT-PCR using *GAPDH* as internal control gene (2^-ΔCt^ method). Statistical analysis has been performed by the two-way analysis of variance to compare multiple mean values through P2-P6-P10. *Abbreviations*: *BM-MSCs* bone marrow-mesenchymal stem cells, *N.S.* no statistically significant difference, *WJ-MSCs* Wharton’s jelly-mesenchymal stem cells

Since replicative cell senescence has been associated with defective proliferative capacity, we investigated whether culture-expanded BM-MSCs are more prone to cellular senescence compared to WJ-MSCs. To this end, we evaluated SA-β-gal activity, telomere length and senescence-associated gene expression through passages, as indices of cell senescence. The number of SA-β-gal-positive cells at P2, P6 and P10 was significantly higher in BM- compared to WJ-MSCs (*P* = 0.0169, *P* = 0.0327 and *P* = 0.0001, respectively) (Table 1). Telomere length was consistently shortened in both BM- and WJ-MSC cultures during passages and the mean relative telomere length of both BM- and WJ-MSCs at P2 was significantly higher compared to P10 (*P* = 0.0208 and *P* = 0.0002, respectively) (Table 1). However, no statistically significant difference was detected between BM- and WJ-MSC cultures in the mean telomere length of cells at P2, P6 and P10 suggesting that the reduced proliferative potential of BM-MSCs compared to WJ-MSCs cannot be attributed to accelerated telomere loss. As regards to senescence-associated gene expression, no significant difference was identified between BM- and WJ-MSCs in *PARG1*, *RB1*, *CDKN1A* and *CDKN2A* gene expression whereas a statistically significant decrease was obtained in *CDKN2B* and *TP53* gene expression in BM-MSCs throughout P2-P6-P10 (*P* < 0.0001 and *P* < 0.0001, respectively) (Fig. 2). Taking together all the above data, and taking into consideration that SA-β-gal activity is neither causative nor prerequisite for manifestation of senescence [20, 21], we conclude that the inferior proliferative potential of BM-MSCs compared to WJ-MSCs cannot be attributed to premature senescence.

**Table 1:** Senescence characteristics and telomere length of WJ-MSCs and BM-MSCs

Passages	SA-β-gal positive cells (%)^a^			Mean telomere length^a^		
	WJ-MSCs	BM-MSCs	*P*	WJ-MSCs	BM-MSCs	*P*
P2	10.28 ± 5.73	25.82 ± 17	0.0169	11,958 ± 1687	10,530 ± 2244	N.S.
P6	15.14 ± 9.73	39.33 ± 22.46	0.0327	10,128 ± 1317	8901 ± 870.9	N.S.
P10	24.09 ± 11	59.14 ± 15.69	0.0001	8529 ± 1619	8212 ± 1486	N.S.

Comparisons in each indicated passage (P; P2, P6, P10) between WJ-MSCs (n = 18) and BM-MSCs (n = 18) have been performed by the nonparametric Mann–Whitney *U* test and the statistically significant *P* values are shown

*Abbreviations: WJ-MSCs* Wharton’s jelly-mesenchymal stem cells, *BM-MSCs* bone marrow-mesenchymal stem cells, *SA-β-gal* senescence-associated β galactosidase, *N.S*. no statistically significant difference

^a^The data are expressed as means ± 1 standard deviation

### Upregulation of cell cycle-related genes in WJ-MSCs

**Fig. 3:**
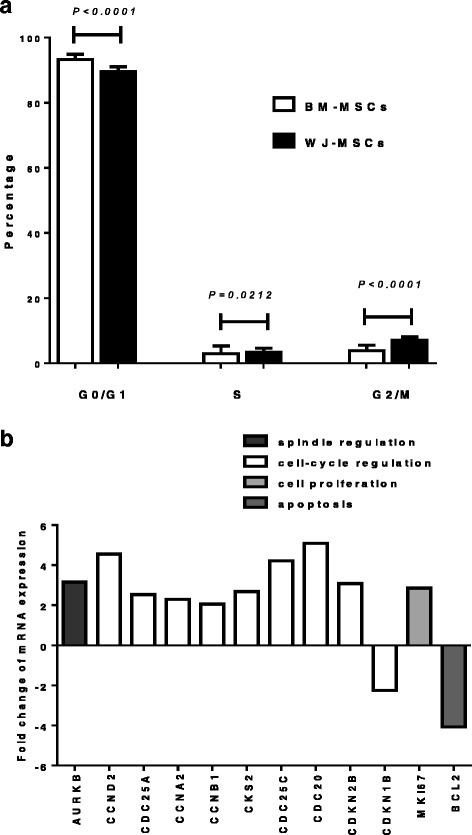
Cell cycle analysis and relative expression of cell cycle-related genes in WJ-MSCs compared to BM-MSCs. **a** BM- and WJ-MSCs from P2, at a confluency of 80–90% were stained by propodium iodide and subsequently cell cycle distribution was analyzed by flow cytometry. *Bars* represent the proportion of cells in each phase of the cell cycle and the values shown are the means ± 1 standard deviation of *n* = 18 independent experiments. **b** The *columns* represent the mean fold change in the expression of genes related to cell cycle in WJ-MSCs (*n* = 6) at P2, compared to the respective BM-MSCs (*n* = 6), using a real-time PCR array. The fold change for each gene between the group of WJ-MSCs and the group of BM-MSCs was calculated with the ΔΔCt method and only genes exhibiting at least a two-fold change are depicted. Differentially expressed genes have been grouped according to their implication in specific cellular process such as spindle regulation, cell cycle regulation, cell proliferation, apoptosis and DNA damage response. *Abbreviations*: *BM-MSCs* bone marrow-mesenchymal stem cells, *P* passage, *WJ-MSCs* Wharton’s jelly-mesenchymal stem cells

We next investigated whether the above described differences in the proliferative characteristics of BM- and WJ-MSCs might be associated with differences in cell cycle distribution of cells in the culture. Indeed, at the representative P2, the proportion of cells in the quiescent G1 phase was significantly higher in BM-MSC cultures compared to WJ-MSCs (93.25 ± 1.63% and 89.54 ± 1.45%, respectively; *P* < 0.0001) (Fig. 3a). In contrast, the percentage of cells in S and G2/M phase in BM-MSC cultures (2.9 ± 2.4% and 3.85 ± 1.66%, respectively) was significantly lower compared to WJ-MSCs (3.34 ± 1.27% and 7.11 ± 0.98%, respectively) (*P* = 0.0212 and *P* < 0.0001, respectively) (Fig. 3a).To probe further the differences in the cell cycle distribution of cells in BM- and WJ-MSC cultures, we subsequently investigated the expression of genes related to cell cycle, by means of a PCR array. By considering as significant an at least two-fold difference in gene expression between WJ- and BM-MSCs [6–8], we found that 13 out of 84 cell cycle-related genes were differentially expressed between the two MSC populations at P2 (Fig. 3b). More specifically, a significant increase in aurora kinase B gene (*AURKB*; FC = 3.1) expression was observed in WJ-MSCs. This gene encodes for a protein that is associated with microtubules during chromosome movement and segregation. Cyclin D2 (*CCND2*) and cell division cycle 25 homologue A (*CDC25A*) gene expression was also upregulated in WJ-MSCs compared to BM-MSCs (FC = 4.5 and FC = 2.5, respectively). Both genes encode for proteins that regulate cell cycle G1/S transition. Furthermore, several genes encoding for cell cycle regulators of G2/M transition were also increased in WJ-MSCs, namely *CCNA2* (FC = 2.3), *CCNB1* (FC = 2.0), a gene encoding for a protein that binds to the catalytic subunit of cyclin-dependent kinases (CDC28 protein kinase regulatory subunit 2, *CKS2*; FC = 2.6), a cyclin-dependent kinase activator (cell division cycle 25C, *CDC25C*; FC = 4.2) and a gene involved in microtubule-associated process (*CDC20*; FC = 5). Moreover, *MKI67* gene, which encodes for a nuclear protein which is associated with cellular proliferation, was also increased in WJ-MSCs (FC = 2.8). In contrast, a significant decrease was obtained in WJ-MSCs compared to BM-MSCs in the expression of the antiapoptotic gene *BCL2* (FC = -4) which mediates delayed transition into S phase as well as in the cyclin-dependent kinase inhibitor-1B (*CDKN1B*; FC = -2.2), which mediates G1 arrest. An upregulation of *CDKN2B* (FC = 3.08) that mediates G1 arrest and upregulation of ataxia-telangiectasia mutated gene (*ATM*; FC = 4.03) that mediates a cell cycle delay after DNA damage, was observed in WJ-MSCs compared to BM-MSCs. Taken together all the above data suggest that WJ-MSCs display mostly an upregulation of genes that stimulate cell proliferation along with a concomitant downregulation of genes related to cell cycle inhibition, compared to BM-MSCs.

### Reduced adipogenic and osteogenic differentiation potential of WJ-MSCs

**Fig. 4:**
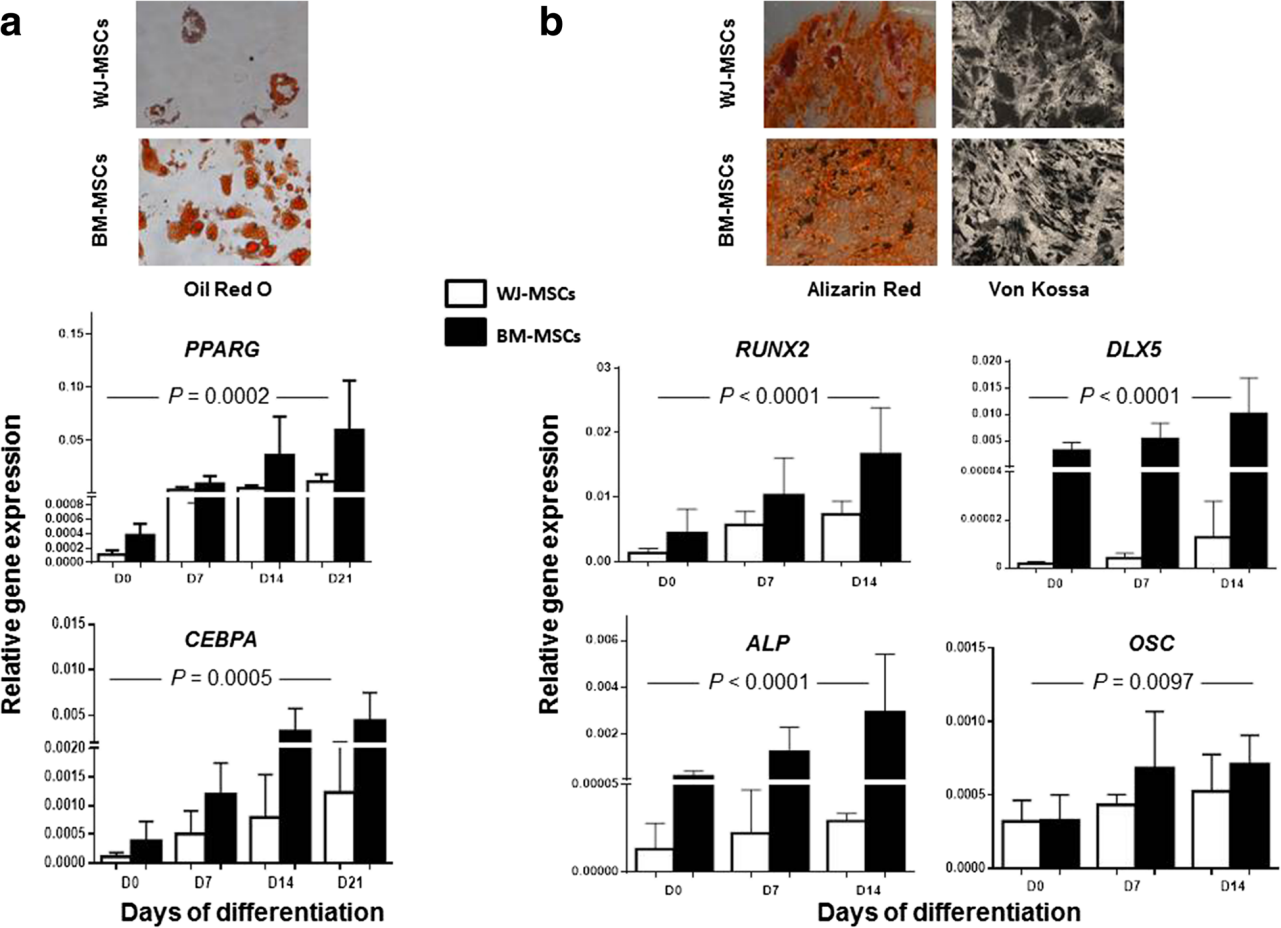
Adipogenic and osteogenic differentiation of WJ-MSCs and BM-MSCs. **a** The adipogenic differentiation of WJ-MSCs and BM-MSCs was assessed by means of Oil Red O staining and real time RT-PCR for the evaluation of the adipocyte-related gene expression. The representative stained samples following adipogenic differentiation depict the inferior potential of WJ-MSCs to differentiate toward adipocytes compared to BM-MSCs. The *bars* in the graphs represent the relative expression (mean + 1 standard deviation) of *PPARG* and *CEBPA* in BM-MSCs (*n* = 18) and WJ-MSCs (*n* = 18) before differentiation induction (day 0) and at different time points during adipogenic differentiation using *GAPDH* as internal control gene (2^-ΔCt^ method). The statistically significant differences, according to the two-way analysis of variance, are depicted. **b** The osteogenic differentiation of WJ-MSCs and BM-MSCs was assessed by means of Alizarin Red and Von Kossa staining and real time RT-PCR for the evaluation of the osteocyte-related gene expression. The representative samples following osteogenic differentiation display similar Alizarin Red and Von Kossa reactions in WJ-MSCs and BM-MSCs. The *bars* in the graphs represent the relative expression (mean + 1 standard deviation) of *RUNX2*, *DLX5*, *ALP* and *OSC* in BM-MSCs (*n* = 18) and WJ-MSCs (*n* = 18) before differentiation induction (day 0) and at different time points during osteogenic differentiation using *GAPDH* as internal control gene (2^-ΔCt^ method). The statistically significant differences, according to the two-way analysis of variance, are depicted. *Abbreviations*: *BM-MSCs* bone marrow-mesenchymal stem cells, *WJ-MSCs* Wharton’s jelly-mesenchymal stem cells

To compare the adipogenic and osteogenic potential of WJ- and BM-MSCs, we induced in parallel adipogenesis and osteogenesis in P2 MSCs from both sources and assessed the specific staining reaction and the expression of adipocyte- and osteocyte-specific genes. WJ-MSCs displayed reduced adipogenic differentiation potential compared to BM-MSCs as indicated by the Oil Red O staining and by the decreased mRNA expression of *PPARG* and *CEBPA* (*P* = 0.0002 and *P* = 0.0005, respectively) (Fig. 4a). WJ-MSCs did not qualitatively differ from BM-MSCs in their capacity to differentiate into osteocytes, as indicated by Alizarin Red and Von Kossa staining (Fig. 4b). However, the mRNA levels of *RUNX2*, *DLX5*, *ALP* and *OSC* were significantly reduced in WJ-MSCs during osteogenic differentiation as compared to BM-MSCs (*P* < 0.0001, *P* < 0.0001, *P* < 0.0001 and *P* < 0.0097, respectively) (Fig. 4b). Interestingly, undifferentiated WJ-MSCs (P2) expressed significantly lower levels of *PPARG*, *CEBPA*, *RUNX2*, *DLX5* and *ALP* compared to BM-MSCs (*P* = 0.0002, *P* = 0.0281, *P* = 0.0141, *P* < 0.0001 and *P* < 0.0001, respectively), suggesting that the WJ-MSCs are less primed toward the adipocytic and osteoblastic lineages compared to BM-MSCs.

### Differential expression of WNT signaling molecules between WJ- and BM-MSCs

**Fig. 5:**
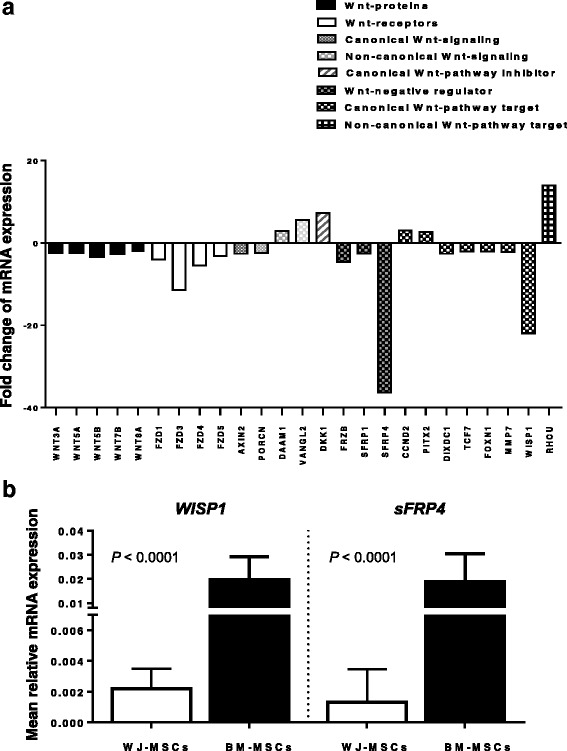
Relative expression of genes related to WNT pathway in WJ-MSCs and BM MSCs. **a** The *columns* represent the mean fold change of genes involved in WNT signaling in P2 WJ-MSCs (*n* = 6) as compared to BM–MSCs (*n* = 6) using a real-time PCR array. Only genes exhibiting at least a two-fold change in their expression are depicted. **b** Validation of the array. The *bars* represent the relative mRNA expression (mean + 1 standard deviation) of *WISP1* and *sFRP4* in WJ-MSCs (*n* = 18) and BM-MSCs (*n* = 18) at P2 using real-time RT-PCR. Values correspond to 2^−ΔCt^ using *GAPDH* as control gene. Comparisons between WJ- and BM-MSCs were performed by the nonparametric Mann-Whitney *U* test and the *P* values are depicted. *Abbreviations*: *BM-MSCs* bone marrow-mesenchymal stem cells, *sFRP4* secreted frizzled related protein 4, *WISP1* WNT1-inducible-signaling pathway protein 1, *WJ-MSCs* Wharton’s jelly-mesenchymal stem cells

The WNT pathway is involved in the differentiation and proliferation process of MSCs [22]. We have therefore evaluated the expression of genes encoding proteins of the WNT pathway in WJ- and BM-MSCs using a PCR array. We found that expression of 25 out of 84 WNT-related genes displayed at least a two-fold difference between WJ- and BM-MSCs (Fig. 5a). Specifically, five genes encoding for WNT proteins were significantly downregulated in WJ-MSCs (*WNT3A*, FC = -2.4; *WNT5A*, FC = -2.3; *WNT5B*, FC = -3.4; *WNT7B*, FC = -2.6; *WNT8A*, FC = -2.2) as well as four genes encoding for WNT receptors (*FZD1*, FC = -4; FZD*3*, FC = -11.4; *FZD4*, FC = -5.4; *FZD5*, FC = -3.2). Two genes implicated in the canonical WNT signaling were downregulated (*AXIN2*, FC = -2.6; porcupine homologue 1 - *PORCN1*, FC = -2.4), whereas two genes involved in the non-canonical WNT signaling were upregulated (disheveled-associated activator of morphogenesis 1 -*DAAM1*, FC = 2.9; Vang-like 2 -*VANGL2*, FC = 5.6) in WJ-MSCs. The gene encoding for the canonical WNT pathway inhibitor dickkopf-1 (*DKK1*) was significantly upregulated (FC = 7.3), while the WNT negative regulators *FRZB*, *sFRP1* and *sFRP4* were decreased (FC = -4.6, FC = -2.5, FC = -36.2, respectively) in WJ-MSCs. The canonical WNT pathway targets DIX domain containing 1 (*DIXDC1*), transcriptional factor-7 (*TCF7*), forkhead box protein N1 (*FOXN1*), matrix metalloproteinase-7 (*MMP7*) and *WISP1* were significantly downregulated in WJ-MSCs (FC = -2.6, FC = -2.1, FC = -2.1, FC = -2.2 and FC = -22, respectively). The expression of *CCND2* and its activator Pituitary homeobox 2 (*PITX2*), which also encode targets of the canonical WNT pathway were significantly increased in WJ-MSCs (FC = 3; and FC = 2.7, respectively). Finally, the expression of the non-canonical WNT target Ras Homolog Family Member U (*RHOU*) gene was significantly increased in WJ-MSCs (FC = 13.9).

Taken together, all the above data suggest that the canonical WNT pathway is downregulated in WJ-MSCs as evidenced by the downmodulation of canonical WNTs, WNT receptors and canonical WNT targets, as well as the upregulation of the WNT/β-catenin signaling inhibitor *DKK1*. We speculate that the reduced expression of the WNT inhibitors/negative regulators *sFRP1*, *FRZB (sFRP3)*, *sFRP4* and *AXIN2* may represent a compensatory phenomenon aiming to upregulate the canonical WNT signaling. The upregulation of genes involved in the non-canonical WNT pathway in WJ-MSCs is also anticipated because the canonical and non-canonical WNT signaling are generally antagonistic [23].

To validate the results obtained from the PCR array, we evaluated the expression of genes with the greatest FC, namely *WISP1* and *sFRP4*, by real-time RT-PCR in P2 WJ-MSCs (n = 18) and BM-MSCs (n = 18). In accordance with the PCR array, the mean relative mRNA expression of both *WISP1* and *sFRP4* was significantly decreased in WJ-MSCs (0.0022 ± 0.0013 and 0.0013 ± 0.002, respectively) compared to BM-MSCs (0.019 ± 0.009 and 0.018 ± 0.01, respectively) (*P* < 0.0001 and *P* < 0.0001, respectively) (Fig. 5b).

### sFRP4 is implicated in the adipogenic differentiation of WJ-MSCs

**Fig. 6:**
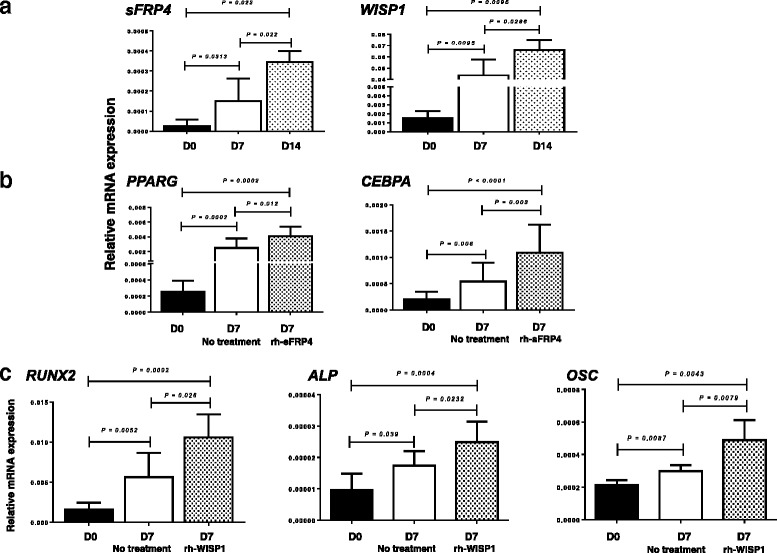
The role of sFRP4 and WISP1 in the adipogenic and osteogenic differentiation of WJ-MSCs. Graph **a** depicts the relative expression (mean + 1 standard deviation) of *sFRP4* and *WISP1* gene expression in WJ-MSCs at different time points during adipogenic and osteogenic differentiation respectively. Graph **b** depicts the relative expression (mean + 1 standard deviation) of the adipocyte-associated genes *PPARG* and *CEBPA* following induction of adipogenic differentiation of WJ-MSCs in the absence or presence of 20nM rh-sFRP4. Graph **c** shows the relative expression (mean + 1 standard deviation) of the osteocyte-associated genes *RUNX2, ALP* and *OSC* following induction of osteogenic differentiation of WJ-MSCs in the absence or presence of 50 ng/ml rh-WISP1. In all cases, evaluation of gene expression was performed by means of real-time RT-PCR using *GAPDH* as control. Comparisons in gene expression between different time points and between treated and untreated cultures were performed by means of the non-parametric Mann–Whitney *U* test. *Abbreviations*: *rh-sFRP4* recombinant human secreted frizzled-related protein 4, *rh-WISP1* recombinant human WNT-induced secreted protein 1, *WJ-MSCs* Wharton’s jelly-mesenchymal stem cells

Secreted-FRP4 is a WNT inhibitor that directly binds WNTs, antagonizing therefore both canonical and non-canonical WNT pathways [24]. It has been reported that rh-sFRP4 may induce adipogenesis of adipose tissue-derived MSCs in vitro [25]. We have therefore investigated whether the profound downregulation of *sFRP4* mRNA expression in WJ-MSCs compared to BM-MSCS (FC = -36.2), might explain the decreased adipocytic differentiation potential of the cells, at least in part. We first evaluated the *sFRP4* gene expression during adipogenic differentiation of WJ-MSCs (n = 5) and we indeed found a gradual statistically significant increase in mRNA levels’ time course (Fig. 6a). Specifically, *sFPR4* mRNA expression at day 14 was significantly higher compared to baseline and day 7 (*P* = 0.022 and *P* = 0.022, respectively).

We next cultured WJ-MSCs in adipocytic medium for 7 days in the absence or presence of 20 nM rh-sFRP4 and evaluated the mRNA levels of the adipogenesis-specific genes *PPARG* and *CEBPA.* As shown in Fig. 6b, at day 7 of adipogenic differentiation, WJ-MSC cultures treated with rh-sFRP4 displayed significant upregulation of the above genes compared to untreated cultures (*P* = 0.012 and *P* = 0.003, respectively) or cultures prior to adipogenic induction (*P* = 0.0002 and *P* < 0.0001, respectively).

### WISP1 is implicated in the osteogenic differentiation of WJ-MSCs

It has been shown that WISP1 promotes the osteogenic differentiation of BM-MSCs and *WISP1* gene expression has been reported to increase during BM-MSC osteogenic differentiation in vitro [26–28]. In accordance with these reports, we also observed a statistically significant upregulation of *WISP1* mRNA expression in WJ-MSCs during osteogenic differentiation (Fig. 6a). Specifically, *WISP1* mRNA expression at day 14 was significantly higher compared to baseline and day 7 (*P* = 0.0095 and *P* = 0.0286, respectively).

To investigate whether the downregulation of *WISP1* mRNA expression in WJ-MSCs compared to BM-MSCs might have a role in the reduced osteogenic differentiation potential of WJ-MSCs, we cultured WJ-MSCs (*n* = 5) in osteogenic medium for 7 days in the absence or presence of 50 ng/ml rh-WISP1 and we next evaluated the mRNA levels of the osteogenesis-specific genes *RUNX2, ALP* and *OSC.* As shown in Fig. 6c, rh-WISP1 treatment resulted in a significant upregulation of these genes compared to untreated WJ-MSCs (*P* = 0.026, *P* = 0.0232 and *P* = 0.0079, respectively) or compared to WJ-MSCs prior to osteogenic induction (*P* = 0.0002, *P* = 0.0004 and *P* = 0.0043, respectively).

### WJ-MSCs exhibit inferior hematopoiesis-supporting potential than BM-MSCs

**Fig. 7:**
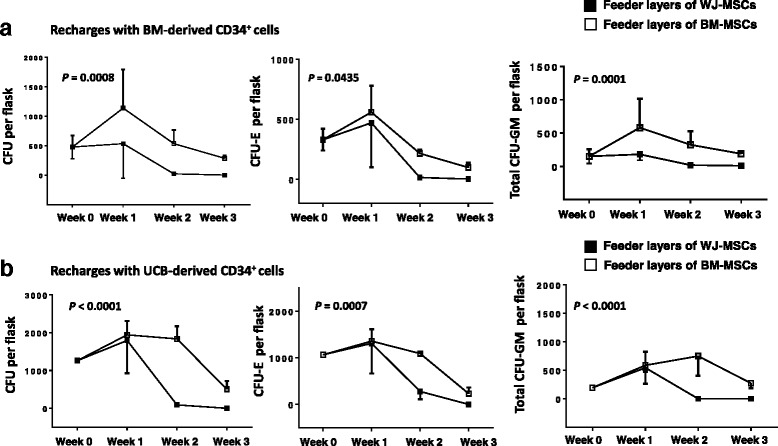
The hematopoiesis-supporting capacity of WJ-MSCs and BM-MSCs. Confluent stromal layers from WJ-MSCs (*n* = 6) and BM-MSCs (*n* = 6) at P2 grown in 25cm^2^ flasks, were irradiated and recharged with normal allogeneic BM-derived (**a**) or UCB-derived (**b**) CD34^+^ cells. At weekly intervals for a total of 3 weeks, cultures were fed by demi-depopulation and the non-adherent cells were assayed for clonogenic progenitor cells in methylcellulose culture medium. The graphs depict the number (mean ± 1 standard deviation) of total colony-forming units (*CFU*), erythroid colony-forming units (*CFU-E*) and total granulocyte-macrophage colony-forming units (*CFU-GM*) time course obtained from the non-adherent cell fraction of recharged WJ-MSC or BM-MSC cultures. Comparison of mean colony numbers over the 3-week culture between WJ- and BM-MSC feeder layers was performed by the two-way analysis of variance and the *P* values are depicted. *Abbreviations*: *BM-MSCs* bone marrow-mesenchymal stem cells, *UCB* umbilical cord blood, *WJ-MSCs* Wharton’s jelly-mesenchymal stem cells

To evaluate the capacity of WJ- and BM-MSCs to support the hematopoietic progenitor cell growth, we assessed the clonogenic potential of the NAC fraction of irradiated (P2) confluent WJ-MSC (*n* = 6) and BM-MSC (*n* = 6) cultures. The frequency of the total CFUs obtained by the NACs was significantly lower in cultures with feeder layers from WJ-MSCs compared to BM-MSCs, irrespectively of the source of the recharging CD34^+^ cells, i.e., BM (*P* = 0.0008) (Fig. 7a) or UCB (*P* < 0.0001) (Fig. 7b). More specifically, the increased CFU numbers in cultures of BM-MSCs compared to the respective of WJ-MSCs, was due to the increased number of both CFU-E and total CFU-GM progenitor cells derived from either BM-CD34^+^ cells (*P* = 0.0435 and *P* = 0.0001, respectively) (Fig. 7a) or UCB-CD34^+^ cells (*P* = 0.0007 and P < 0.0001, respectively) (Fig. 7b). Interestingly, both WJ- and BM-MSC feeder layers exhibited superior hematopoiesis-supporting capacity when recharged with UCB-CD34^+^ cells than BM-CD34^+^ cells, as indicated by the evaluation of the total CFU numbers (*P* < 0.0001 and *P* < 0.0001, respectively).

To probe further the hematopoiesis-supporting potential of WJ- and BM-MSCs, we analyzed several common hematopoietic cytokines, namely FLT3L, SDF-1α, G-SCF and GM-CSF in P2, P6 and P10 supernatants from WJ-MSC (*n* = 18) and BM-MSC (*n* = 18) cultures. As shown in Table 2, FLT3L levels did not differ significantly between WJ- and BM-MSC cultures whereas SDF-1α levels were significantly decreased in WJ- compared to BM-MSC culture supernatants throughout passages (*P* = 0.0012). G-CSF and GM-CSF were detectable in WJ-MSC supernatants through passages but were undetectable in the respective BM-MSC cultures (*P* < 0.0001 and *P* < 0.0001, respectively). These data suggest that the inferior hematopoiesis-supporting capacity of WJ-MSCs compared to BM-MSCs is unlikely to be associated with impaired production of hematopoiesis-inducing cytokines, but may be associated with the lower production of SDF-1α, at least in part.

**Table 2:** Cytokines in MSC culture supernatants

Cytokines^a^	WJ-MSC culture supernatants (n = 18)			BM-MSC culture supernatants (n = 18)			
	P2	P6	P10	P2	P6	P10	*P* value
FLT3L (ng/mL)	13.79 ± 0.75	14.35 ± 1.24	13.74 ± 0.87	13.77 ± 0.96	14.70 ± 6 2.33	13.43 ± 0.45	N.S.
SDF1-α (ng/mL)	1189.00 ± 380.40	1159.00 ± 401.00	1032.00 ± 269.00	2074.00 ± 1247.00	1607.00 ± 698.00	1369.00 ± 420.60	0.0012
G-CSF (ng/mL)	108.30 ± 100.70	578.00 ± 409.80	388.00 ± 360.00	N.D.	N.D.	N.D	<0.0001
GM-CSF (ng/mL)	12.19 ± 9.46	145.20 ± 135.60	37.70 ± 13.58	N.D.	N.D.	N.D.	0.0002

Cytokine measurements have been performed by means of an enzyme-linked immunosorbent assay (ELISA). Comparisons in cytokine levels between WJ-MSCs and BM-MSC supernatants through passages (P) P2-P6-P10, were performed by two-way analysis of variance and the statistically significant *P* values are shown

*Abbreviations: WJ-MSCs* Wharton’s jelly-mesenchymal stem cells, *BM-MSCs* bone marrow-derived mesenchymal stem cells, *P* passage, *FLT3L* FMS-like tyrosine kinase 3 ligand, *SDF-1α* stromal cell-derived factor-1α, *G-CSF* granulocyte colony-stimulating factor, *GM-CSF* granulocyte-macrophage colony -stimulating factor, *N.S*. no statistically significant difference, *N.D*. not detected

^a^The data are expressed as means ± 1 standard deviation

## Discussion

WJ-derived MSCs have gained much attention over the last years for potential use in cell-based therapies, since they can be easily isolated without any ethical concerns from a tissue which is discarded after birth [3]. In the present study, we have performed a rigorous side-by-side comparison of WJ-MSCs with BM-MSCs, which represents the most extensively studied MSC population. We even excluded the possible contamination of WJ-MSCs by diverse MSC populations from different UC regions, i.e., perivascular and sub-amnion, by stripping the UC vessels before cutting the WJ into pieces and use them as explants for culture establishment. Various biological properties of the two MSC populations were comparatively studied in parallel experiments, including the expression of cell cycle and WNT pathway-related genes, which have never been studied before. The involvement of WNT pathway-related molecules in the differentiation capacity of WJ-MSCs were also studied for the first time.

Ex vivo expanded WJ-MSCs showed similar fibroblast-like morphology and immunophenotypic characteristics compared to BM-MSCs. However, the growth rate of WJ-MSCs over passages was significantly increased compared to BM-MSCs, as was demonstrated by the PD time through passages and the metabolic activity of cells at a representative passage (P2). Our data on the survival characteristics of MSCs suggest that the differential growth of the two MSC populations cannot be attributed to differences in the rate of apoptotic cell death but may be associated to the increased proliferation of WJ-MSCs, at least in part. Indeed, significant higher proportion of WJ-MSCs was found in S and G2/M phase of cell cycle in flow cytometric analysis, compared to BM-MSCs, and this finding was associated with alterations in the expression of genes involved in G1/S and G2/M transition. Specifically, an upregulation of *CCND2*, *CDC25A*, *CCNA2*, *CCNB1*, *CDC28*, *CKS2*, *CDC25C*, *CDC20* and *AURKB* was found in WJ-MSCs compared to BM-MSCs that was associated with downregulation of the anti-apoptotic gene *BCL2*, which mediates delayed transition into S phase, and downregulation of *CDKN1B*, which is implicated in G1 arrest. To the best of our knowledge, comparative analysis of cell cycle-related genes between WJ-MSCs and BM-MSCs has not been previously reported and the results give an explanation for the increased growth rate of the WJ-MSCs in comparison to their BM counterparts.

Since replicative senescence has been associated with defective cellular proliferative capacity, we investigated whether the inferior growth of BM-MSCs throughout culture might be due to increased propensity to cell senescence compared to WJ-MSCs. However, the expression of three cell cycle inhibitors which are usually upregulated in senescent cells, namely *RB1*, *CDKN1A*, *CDKN2A*, as well as the expression of the senescence associated-gene *PARG1* known to increase during passages [29, 30], did not differ significantly between the two MSC populations at P2-P6-P10. *TP53* was found overexpressed in WJ-MSCs compared to BM-MSCs. However, *TP53* is not solely associated to cell senescence but also functions as a guard of the genome by inhibiting the cell cycle and therefore allowing the time for repair of potential DNA damage [31]. Based on the increased proliferation potential of WJ-MSCs, one may hypothesize that the upregulation of *TP53* may act a safety mechanism, ensuring that these cells will not accumulate genomic instability during successive divisions.

Telomere length has been recognized as the main indicator of MSC replicative senescence [21, 32]. The mean telomere length did not differ significantly between WJ- and BM-MSCs through passages, suggesting further that cell senescence is unlikely to account for the inferior proliferative potential of BM-MSCs. In contrast to the aforementioned observations, the number of SA-β-gal-positive cells was significantly higher in BM-MSC cultures compared to WJ-MSCs throughout passages. Nevertheless, SA-β-gal activity, although associated, is not completely specific for senescence [33, 34]. Instead, it can give false-positive results when used as the sole method to identify senescence and should therefore be interpreted in combination with other biomarkers [33, 34]. However, given that the expression of SA-β-gal has been mostly associated with a non-proliferative state [34], the increased proportion of SA-βgal-positive cells, may have a role in the decreased proliferative characteristics of BM- compared to WJ-MSCs.

The use of MSCs in cell-based therapies may require large-scale ex vivo expansion. To investigate whether long-term culture may influence the genetic stability of WJ-MSCs, we performed cytogenetic analysis of WJ- and BM-MSCs through passages. Clonal cytogenetic aberrations were detected in WJ-MSCs from 2/18 (11.11%) samples [del(1)(p34) and add(2)(p25.3)] and in BM-MSCs from 3/18 (16.67%) samples (trisomy 5). Notably in all cases, MSCs showed progressive growth arrest and eventually entered senescence. Our findings are in line with previous reports suggesting that BM-MSCs may occasionally develop genetic aberrations after long-term culture, without evidence of malignant transformation whatsoever [35–37]. As regards WJ-MSCs, contradictory results have been reported so far for the genomic stability of UC-derived MSCs [38–42]. The inconsistencies are probably related to the variability of MSC populations, culture conditions and methodologies (classical karyotype, FISH or comparative genomic hybridization array). Nevertheless, on the basis of our findings, BM-MSCs and WJ-MSCs develop chromosomal aberrations at the same rate when expanded under the same experimental conditions and karyotypic analysis of culture-expanded MSCs is reasonable before any application of cells in the clinic.

With regards to the ex vivo differentiation potential, WJ-MSCs displayed inferior adipogenic capacity compared to BM-MSCs, as evidenced by the Oil Red O staining and the expression of adipogenesis-specific genes *PPARG* and *CEBPA.* The WJ-MSCs also displayed reduced osteogenic potential compared to BM-MSCs, as evidenced by the lower expression of the osteogenesis-related genes *RUNX2*, *DLX5*, *ALP* and *OSC* despite the similar Von Kossa and Alizarin Red staining. The above findings are in line with previous reports indicating reduced differentiation capacity of WJ-MSCs toward adipocytes and osteocytes [43–45]. In our study, we also showed decreased expression of osteogenesis- and adipogenesis-related genes in undifferentiated WJ-MSCs at P2 compared to BM-MSCs, suggesting an inferior lineage priming of WJ-MSCs to osteoblastic and adipocytic lineages.

Because mechanisms accounting for the weak differentiation potential of WJ-MSCs toward adipocytes and osteocytes have not been elucidated thus far and based on the established role of the WNT signaling pathway in MSC differentiation [46–49], we comparatively investigated the expression profile of genes associated with this pathway in BM- and WJ-MSCs. Twenty-five out of 84 genes were found to be differentially expressed between the two cell populations. Although the functional significance of these differences remains to be fully elucidated, we speculate that the differential expression of the WNT pathway-associated molecules has probably a role in the inferior osteogenic and adipogenic potential of WJ-MSCs compared to BM-MSCs. Indeed, *WNT3A*, -*5A* and -*7B* known to promote osteogenesis [46, 47] were downregulated, whereas the canonical WNT pathway negative regulator *DKK1*, known to inhibit osteogenesis [46], was upregulated in WJ-MSCs. Furthermore, based on the finding of significant downregulation of *WISP1* in WJ- compared to BM-MSCs and also on previously reported data on the role of this molecule in osteoblastic differentiation in vitro and osteogenesis in vivo [26, 27], we studied its significance in the osteogenic differentiation of WJ-MSCs. The upregulation of *WISP1* expression during the osteogenic differentiation of WJ-MSCs in association with the finding that treatment with rh-WISP1 resulted in induction of the osteogenic potential of WJ-MSCs, as was evidenced by the upregulation of *RUNX2*, *ALP* and *OSC* expression, corroborates the assumption that the downregulation of *WISP1* in WJ-MSCs compared to BM-MSCs is probably implicated in their inferior differentiation capacity toward the osteoblastic lineage.

The implication of WNT signaling pathway in the adipogenic differentiation of MSCs has been previously reported [48, 49]. In this context, and because *sFRP4*, a molecule known to be implicated in adipogenesis [25, 50], was found significantly downregulated in WJ-MSCs compared to BM-MSCs, we investigated the potential role of *sFRP4* in the adipogenic differentiation of WJ-MSCs. The increase of *sFRP4* mRNA during the adipogenic differentiation of WJ-MSCs in association with the finding that treatment with rh-sFRP4 resulted in induction of the adipogenic potential of WJ-MSCs, as was indicated by the upregulation of *PPARG* and *CEBPA* expression, favors the hypothesis that the downregulation of *sFRP4* in WJ-MSCs compared to BM-MSCs is probably implicated in their inferior differentiation capacity toward adipocytes. The involvement of *WISP1* and *sFRP4* on the differentiation potential of WJ-MSCs provide novel insights on the role of WNT signaling in this MSC population.

Limited data are currently available on the hematopoiesis-supporting capacity of WJ-MSCs compared to BM-MSCs [51, 52]. To probe this issue, we recharged in parallel WJ- and BM-MSC stromal layers with the same batch of UCB- or BM-derived CD34^+^ cells and subsequently evaluated the capacity of the stromal cells to support the clonogenic potential of the supernatant CD34^+^ cells, over 3 weeks. We found that feeder layers derived from WJ-MSCs displayed an inferior potential to support myeloid and erythroid colony formation by the supernatant hematopoietic cells, compared to BM-MSCs. This observation corroborates a previous report [51], whereas it contradicts another study which detected no difference in the hematopoiesis-supporting function of UC- and BM-MSCs [52]. This controversy might be attributed, at least in part, to the fact that in the latter study MSCs from the whole UC were used as feeder layers.

The observed differences in the hematopoiesis-supporting capacity of WJ- compared to BM-MSCs might be due, at least in part, to the different cytokine production. In line with a previous report [53], we have also shown that WJ-MSCs secrete lower SDF-1α and higher G-CSF and GM-CSF amounts, compared to BM-MSCs. We assume that the increased levels of G-CSF and GM-CSF may not be able to functionally counterbalance the decreased production of SDF-1α by the WJ-MSCs [54, 55]. The differential expression of the WNT pathway-related molecules between WJ- and BM-MSCs may also have a role in the different hematopoiesis-supporting function of these two MSC populations. For example, the canonical WNT pathway, which was downregulated in WJ-MSCs, has been reported to display a positive effect [56], whereas the canonical pathway inhibitor DKK1, which was upregulated in WJ-MSCs, has been reported to exert a negative effect on the hematopoietic progenitor cell growth [57]. Interestingly, both WJ- and BM-MSCs were less effective in supporting the growth of the BM-derived than the UCB-derived CD34^+^ cells probably because the BM hematopoietic stem/progenitor cell fraction contains less primitive and fewer clonogenic cells compared to UCB [58].

## Conclusions

Overall, we have performed a rigorous, head-to-head comparison of ex vivo expanded WJ-MSCs and BM-MSCs and have provided insights in their immunophenotypic, survival, proliferative and cytogenetic characteristics, their differentiation potential and their capacity to support hematopoiesis. We have provided, for the first time, evidence for differential expression of cell cycle and WNT pathway-related molecules that may significantly contribute to the observed differences in the biological properties of the two MSC populations. We have also pointed out the prominent role of the WNT pathway-related molecules *WISP1* and *sFRP4* in the osteogenic and adipogenic differentiation potential of WJ-MSCs. These data contribute to the better characterization of WJ-MSCs and BM-MSCs in view of their use in the clinic and even encourage the probability of engineering the WNT pathway in WJ-MSCs for specific clinical applications.

## Abbreviations

7-AAD: 7-aminoactinomycin D
ALP: Alkaline phosphatase
BM: Bone marrow
CDKN: Cyclin-dependent kinase
CEBPA: CCAAT/enhancer-binding protein alpha
CFU: Colony-forming units
DLX5: Distal-less homeobox protein 5
FC: Fold change
FLT3L: FMS-like tyrosine kinase 3 ligand
GAPDH: Glyceraldehyde-3-phosphate dehydrogenase
G-CSF: Granulocyte colony-stimulating factor
GM-CSF: Granulocyte-macrophage colony-stimulating factor
MSC: Mesenchymal stem cells
MTT: 3-[4,5-dimethylthiazol-2-yl]-2,5 diphenyl tetrazolium bromide
NACs: Non-adherent cells
OSC: Osteocalcin
P: Passage
PARG1: Phosphate-associated RhoGAP protein-tyrosine
PD: Population doubling
PPARG: Peroxisome proliferator activated receptor-γ
RB1: Retinoblastoma
RT-PCR: Real-time reverse transcription polymerase chain reaction
RUNX2: Runt-related transcription factor 2
SA-β-gal: Senescence-associated β-galactosidase
SDF-1α: Stromal cell-derived factor-1α
sFRP4: Secreted frizzled related protein 4
UC: Umbilical cord
UCB: Umbilical cord blood
WISP1: WNT1-inducible-signaling pathway protein 1
WJ: Wharton’s jelly
